# Supplementary material for: ORCH: many analyses, one merge—a deterministic multi-agent orchestrator for discrete-choice reasoning with EMA-guided routing
Source: Front Artif Intell. 2026 Feb 2;9:1748735. doi: 10.3389/frai.2026.1748735 (PMC12907423; doi:10.3389/frai.2026.1748735)
Supplement: Supplementary file 1 [file Data_Sheet_1.docx]

**Appendix**  A compact worked example from input to final output

Step 1 — Question decomposition (dispatcher: agent-openai)

ORCH first asks a dispatcher agent to rewrite the original multiple-choice item into three guided sub-questions.

These sub-questions are designed to be complementary (concept check → option elimination → verification),

so that different agents can work in parallel on different aspects of the same problem.

CALL agent-openai -> (latency=1423ms, http=200, tok_in=186, tok_out=45)

TEXT:

1. What is the inverse element of a group, and how is it defined?

2. Which options do not meet the definition of the inverse element of a group?

3. How to verify whether each option satisfies the condition of an inverse element?

--------------------------------------------------------------------

Step 2 — Parallel analysis (three agents, one sub-question each)

ORCH assigns the three sub-questions to three different base agents.

Each agent produces an independent, structured reasoning trace (≤500 chars enforced in code) and a tentative A/B/C/D.

This parallelization is intended to increase diversity and reduce single-agent blind spots.

Step 2-1 — Analysis agent #1 (agent-xai)

CALL agent-xai -> (latency=3311ms, http=200, tok_in=201, tok_out=286)

TEXT:

【Key Points】

1. Definition of a group: (Z,*) is a group if for all a∈Z there exists an inverse element a' such that a*a'=e, where e is the identity element.

2. Definition of operation: a*b = a+b+1.

3. Determining the identity element e: Let e ​​be the identity element. Then for any a∈Z, a*e = a, i.e., a+e+1 = a, solving for e gives e = -1.

4. Defining the inverse element: Let a' be the inverse element of a. Then a*a' = e, i.e., a+a'+1 = -1, solving for a' gives a' = -a-2.

5. Verifying the options:

- A. 0: a*0 = a+0+1 = a+1 ≠ -1, not satisfying the inverse element condition.

- B. -2: a*(-2) = a-2+1 = a-1 ≠ -1, not satisfying the inverse element condition.

- C. a-2: a*(a-2) = a+(a-2)+1 = 2a-1 ≠ -1, not satisfying the inverse element condition.

- D. (2+a)*-1: a*((2+a)*-1) = a+(-(2+a))+1 = a-2-a+1 = -1, satisfying the inverse condition.

Tentative Answer: D

--------------------------------------------------------------------------------

Step 2-2 — Analysis agent #2 (agent-openai)

CALL agent-openai -> (latency=9852ms, http=200, tok_in=200, tok_out=528)

TEXT:

【Key Points】

1. **Definition of a Group**:

- A group is a set G and a binary operation *, satisfying closure, associativity, existence of an identity element, and existence of an inverse element.

2. **Definition of the Operation**:

- In this problem, the operation is defined as a*b = a + b + 1.

3. **Determination of the Identity Element**:

- To find the identity element e such that for any a ∈ Z, a*e = a.

- By the definition of the operation, a*e = a + e + 1 = a. - Solving for e, we get e = -1, therefore the identity element is -1.

4. **Definition of the inverse element**:

- For any element a, the inverse element b must satisfy a*b = e, i.e., a*b = -1.

- According to the definition of operation, a*b = a + b + 1 = -1.

- Solving for b, we get b = -2 - a.

5. **Option Analysis**:

- A. 0: Does not meet the definition of the inverse element.

- B. -2: Does not meet the definition of the inverse element because the inverse element should be -a-2.

- C. a-2: Meets the form of the inverse element, but it needs to be verified whether a*(a-2) = -1.

- D. (2+a)*-1: The form is complex, and it needs to be verified whether it meets the condition of the inverse element.

6. **Verification of Option C**:

- Calculate a*(a-2):

- a*(a-2) = a + (a-2) + 1 = 2a - 1. - Let 2a - 1 = -1, solving for a gives a = 0, indicating that C holds true in a specific case, but is not universally applicable.

7. **Verification of Option D**:

- Calculate a*((2+a)*-1):

- Let b = (2+a)*-1, calculate a*b = a + b + 1.

- Substituting b, we get a + (2+a)*-1 + 1 = a - (2+a) + 1 = -1.

- This shows that D satisfies the condition for an inverse element.

【Exclusion Logic】

- A and B do not meet the definition of an inverse element.

- C holds true in a specific case, but not for all a.

- D meets the definition of an inverse element and applies to all a.

Provisional Answer: D

--------------------------------------------------------------------------------

Step 2-3 — Analysis agent #3 (agent-deepseek)

CALL agent-deepseek -> (latency=15910ms, http=200, tok_in=180, tok_out=376)

TEXT:

【Key Points】

1. Definition of group inverse: For any \( a \in G \), there exists \( a^{-1} \) such that \( a * a^{-1} = e \), where \( e \) is the identity element.

2. First find the identity element \( e \): From \( a * e = a + e + 1 = a \), we get \( e = -1 \).

3. Let the inverse of \( a \) be \( x \), then \( a * x = a + x + 1 = -1 \) ⇒ \( x = -a - 2 \).

4. Verify the options:

- A. 0: Only true if \( -a - 2 = 0 \) i.e., \( a = -2 \), not universally applicable.

- B. -2: Only true if \( -a - 2 = -2 \) i.e., \( a = 0 \), not universally applicable.

- C. \( a - 2 \): Contrary to \( -a - 2 \), always false (unless \( ​​a = 0 \)).

- D. \( (2 + a)^{-1} \): The sign is the reciprocal, irrelevant to the definition of the operation, and not always true in \( \mathbb{Z} \).

5. Elements that do not conform to the definition of an inverse element must be invalid for all \( a \): A, B, C, and D are not identically equal to \( -a - 2 \), but the question asks "which do not conform", meaning they cannot be used as a universal inverse formula. A, B, C, and D all fail to meet the definition, but if one must be chosen that least violates the definition of a group inverse (i.e., is completely wrong), D is clearly wrong because its meaning (reciprocal) is irrelevant to the operation.

Tentative answer: D

----------------------------------------------------------------------------------------

Step 3 — Merge (merger: agent-openai)

ORCH then calls a merge agent that is only allowed to output a single letter (A/B/C/D).

The merge agent reads the three analysis traces and emits the final ORCH decision for this question.

CALL agent-openai -> (latency=763ms, http=200, tok_in=1114, tok_out=1)

TEXT:D

---------------------------------------------------------------------------------------

Step 4 — Single-shot baselines (one call per model, no decomposition, no merge)

For comparison, each baseline agent is queried once on the original question and must return only A/B/C/D.

These baseline calls are independent from ORCH’s multi-step pipeline.

CALL agent-openai -> (latency=846ms, http=200, tok_in=159, tok_out=1)

TEXT:C

----------------------------------------------------------------------------------------

CALL agent-deepseek -> (latency=990ms, http=200, tok_in=152, tok_out=1)

TEXT:B

----------------------------------------------------------------------------------------

CALL agent-xai -> (latency=578ms, http=200, tok_in=156, tok_out=1)

TEXT:D

----------------------------------------------------------------------------------------

Step 5 — Final per-item record (gold label + ORCH + baselines + vote + latency)

The evaluation script records: gold answer, ORCH decision, baseline decisions, (optional) majority vote, and ORCH latency.

[abstract_algebra Q3/30] GOLD=D | ORCH=D | OPENAI=C | DEEPSEEK=B | XAI=D | VOTE=C | lat_ORCH=18104ms
